# A protein risk score for all-cause and respiratory-specific mortality in non-Hispanic white and African American individuals who smoke

**DOI:** 10.1038/s41598-024-71714-7

**Published:** 2024-09-04

**Authors:** Matthew Moll, Katherine A. Pratte, Catherine L. Debban, Congjian Liu, Steven A. Belinsky, Maria Picchi, Iain Konigsberg, Courtney Tern, Heena Rijhwani, Brian D. Hobbs, Edwin K. Silverman, Yohannes Tesfaigzi, Stephen S. Rich, Ani Manichaikul, Jerome I. Rotter, Russel P. Bowler, Michael H. Cho

**Affiliations:** 1 Channing Division of Network Medicine, Department of Medicine, Brigham and Women’s Hospital, 181 Longwood Ave, Boston, MA 02115 USA; 2 Division of Pulmonary and Critical Care Medicine, Department of Medicine, Brigham and Women’s Hospital, Boston, MA 02115 USA; 3 Division of Pulmonary, Critical Care, Sleep and Allergy, Veterans Affairs Boston Healthcare System, West Roxbury, MA 02123 USA; 4 Department of Biostatistics, National Jewish Health, Denver, CO 80206 USA; 5 Center for Public Health Genomics, University of Virginia School of Medicine, Box 800717, Charlottesville, VA 22908 USA; 6 University of New Mexico Comprehensive Cancer Center, Albuquerque, NM USA; 7 Lovelace Biomedical Research Institute, Albuquerque, NM USA; 8 Department of Biomedical Informatics, University of Colorado Anschutz Medical Campus, Colorado, Aurora USA; 9 Regeneron Pharmaceuticals, Tarrytown, NY 10591 USA; 10 The Institute for Translational Genomics and Population Sciences, Department of Pediatrics, The Lundquist Institute for Biomedical Innovation at Harbor-UCLA Medical Center, Torrance, CA 90509 USA; 11 Division of Pulmonary, Critical Care and Sleep Medicine, National Jewish Health, Denver, CO 80206 USA; 12 Harvard Medical School, Boston, USA

**Keywords:** Predictive medicine, Predictive markers, Chronic obstructive pulmonary disease

## Abstract

Protein biomarkers are associated with mortality in cardiovascular disease, but their effect on predicting respiratory and all-cause mortality is not clear. We tested whether a protein risk score (protRS) can improve prediction of all-cause mortality over clinical risk factors in smokers. We utilized smoking-enriched (COPDGene, LSC, SPIROMICS) and general population-based (MESA) cohorts with SomaScan proteomic and mortality data. We split COPDGene into training and testing sets (50:50) and developed a protRS based on respiratory mortality effect size and parsimony. We tested multivariable associations of the protRS with all-cause, respiratory, and cardiovascular mortality, and performed meta-analysis, area-under-the-curve (AUC), and network analyses. We included 2232 participants. In COPDGene, a penalized regression-based protRS was most highly associated with respiratory mortality (OR 9.2) and parsimonious (15 proteins). This protRS was associated with all-cause mortality (random effects HR 1.79 [95% CI 1.31–2.43]). Adding the protRS to clinical covariates improved all-cause mortality prediction in COPDGene (AUC 0.87 vs 0.82) and SPIROMICS (0.74 vs 0.6), but not in LSC and MESA. Protein–protein interaction network analyses implicate cytokine signaling, innate immune responses, and extracellular matrix turnover. A blood-based protein risk score predicts all-cause and respiratory mortality, identifies potential drivers of mortality, and demonstrates heterogeneity in effects amongst cohorts.

## Introduction

Chronic obstructive pulmonary disease (COPD), characterized by persistent airflow limitation, is a leading cause of mortality worldwide^1^. This disease is heterogeneous with respect to respiratory symptoms, emphysema, airway pathology, exacerbations, and mortality^2–4^. Identifying COPD individuals at high risk of mortality can help clinicians tailor therapies, monitor for progression, and aid in timely lung transplant referral^5–7^.

Multiple mortality prediction models in COPD have been developed. The body-mass index, obstruction, dyspnea, exercise capacity (BODE) index predicts 4-year mortality in COPD patients^8^. Other scores have performed similarly to the BODE index^9,10^ and adding CT imaging variables added statistically significant yet small increments in predictive performance when added to the BODE index^11^. These clinical prediction models have limitations: models are cumbersome and critical variables are often difficult to obtain in a primary care setting. For example, spirometry and 6-min walk distances, arguably the most important clinical predictors of mortality^11,12^, are challenging to obtain during a short outpatient visit. Further, the existing models focus on those with COPD, yet emerging data have described that individuals with normal spirometry and preserved ratio with impaired spirometry (PRISm) can progress to having moderate-to-severe airflow obstruction and are at risk for symptoms, emphysema, exacerbations, and death^13,14^.

Proteomics are appealing for mortality prediction. Protein-based biomarkers have the potential to identify rational drug targets and drug repurposing candidates. In addition, proteomics have demonstrated success in predicting all-cause mortality above traditional risk factors^15,16^. However, these studies have not focused specifically on persons who smoked and those selected for COPD. Whether a protein-based risk score can predict all-cause and respiratory mortality in a cohort of smokers is unknown. We hypothesized that a protein risk score (protRS) could improve prediction of all-cause and respiratory mortality over traditional clinical risk factors in multiple cohorts enriched for persons who smoke.

## Methods

### Study populations

All study participants and/or the legal guardian(s) of dead participants provided written informed consent and institutional review board (IRB) approval was obtained at each institution. This research complies with the Declaration of Helsinki. All experimental protocols and the current analysis were approved by the Brigham and Women’s IRB protocol (#2007P000554) or local IRB, as appropriate. In the current study, we included only individuals with SomaScan and mortality data, the details for which are in the supplementary appendix.

### Smoking cohorts

#### COPDGene

The Genetic Epidemiology of COPD (COPDGene) study^17^ recruited 10,198 non-Hispanic white (NHW) and African American (AA) individuals with ≥ 10 pack-years of smoking, aged 45–80 years. Baseline demographic, spirometry, computed tomography (CT) imaging data, and whole blood samples were collected. We included individuals with proteomic and mortality data at the time of study enrollment.

#### SPIROMICS

The SubPopulations and Intermediate Outcome Measures in COPD Study (SPIROMICS)^18^ recruited NHW and AA individuals aged 40 to 80 years with smoking history ≥ 20 pack-years. Recruitment included non-smokers (< 1 pack-year) with FEV_1_/FVC > 0.7 and FVC > LLN (Stratum 1), or history of smoking > 20 pack-years and divided into strata based on spirometry: Stratum 2: without COPD (FEV_1_/FVC > 0.7 and FVC > LLN); Stratum 3: mild-to-moderate COPD (FEV_1_/FVC < 0.7 and FEV1 > 50% predicted); and Stratum 4: severe COPD (FEV_1_/FVC < 0.7 and FEV_1_ < 50% predicted.

#### Lovelace smokers’ cohort

The Lovelace Smokers’ Cohort (LSC)^19,20^ recruited participants from the Albuquerque, New Mexico metropolitan area aged 40–75 years with 10 or more pack-years of smoking who were able to understand English. Anthropometric, spirometry, and proteomic data were collected at the baseline visit. Participants were followed for a median of 6 years.

### General population cohorts

#### MESA

The Multi-Ethnic Study of Atherosclerosis (MESA)^21^ is a prospective U.S.-based study of community-dwelling adults originally designed to examine subclinical cardiovascular disease. MESA participants were free of clinical cardiovascular disease at baseline.

### Statistical analyses

#### Overview of study design

**Fig. 1:**
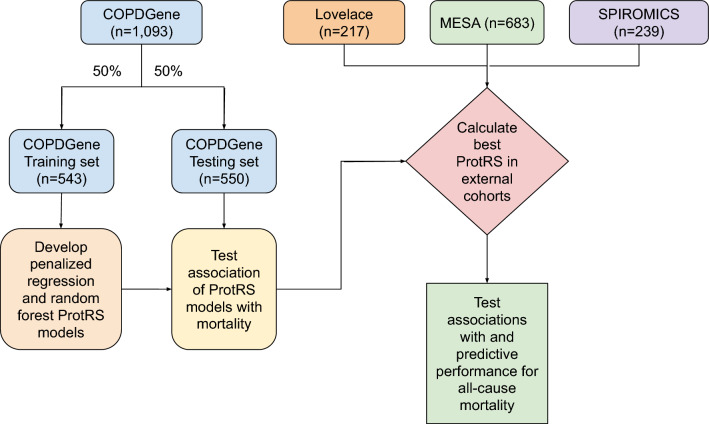
Schematic of study design. COPDGene, Genetic Epidemiology of COPD study. MESA, Multi-Ethnic Study of Atherosclerosis. SPIROMICS, SubPopulations and InteRmediate Outcomes Measures in COPD Study. ProtRS, Protein risk score.

A schematic of our study design is shown in Fig. 1. As COPDGene was our largest study, we split COPDGene samples into training and testing samples. Using the training sample, we developed multiple proteomic models to predict mortality, and tested performance in the COPDGene testing sample. Based on testing in COPDGene, we selected a single model for external replication in LSC, MESA, and SPIROMICS.

#### Development of a protein risk score

We randomly split the COPDGene dataset into training and testing samples (50:50). Using the training sample, we constructed four models: (1) least absolute shrinkage selector operator (LASSO), (2) adaptive LASSO (ada-LASSO), (3) Random Forest, (4) Random survival forest (RSF). We used the glmnet R package to calculate LASSO and ada-LASSO scores, performing tenfold cross validation to optimize the c-index. For random forest-based algorithms, we determined the combination of trees and nodes that yielded the lowest mean squared error (MSE) in the training sample for predicting mortality using the randomForest R package (500 trees and 6 nodes). We used the random survival forest (RSF) R package to construct RSF models to predict time-to-death using 1000 trees and 5 nodes. All scores were rank normalized prior to statistical analysis. We tested the association of each proteomic risk model with time-to-death (see Outcomes, models, and specifications) in the COPDGene testing set, and selected the protein risk score (protRS) based on the largest observed effect size and model parsimony.

As we used COPDGene, MESA, and SPIROMICS SomaScan 1.3 K and LSC SomaScan 5 K data, we performed a sensitivity analysis to determine whether a protRS derived in the 1.3 K data was transferable to the 5 K data. Using COPDGene samples with 1.3 K proteomic data at baseline and 5 K data at the 5-year follow up visit, we calculated the protRS at both time points and tested the correlation of the 5 K score with the original 1.3 K SomaScan score using the Pearson correlation coefficient. We further tested the multivariable association of the 5 K score with time-to-death as described below.

#### Outcomes, models, and specifications

The primary outcome was time-to-death (i.e. all-cause mortality), which was available in all cohorts. Cox^22^ regression models were constructed to evaluate the association between the protRS and time-to-death. We tested the proportional hazards assumption using Schoenfeld residual plots and tests. We tested for model miscalibration using a modified D’Agostino Nam test^23^. We referenced the transparent reporting of a multivariable prediction model for individual prognosis or diagnosis (TRIPOD) reporting standards^24^ to ensure transparent reporting of our prediction model. In multivariable regression analyses, we adjusted for potential confounders based on clinician input and BODE variables, including age, sex, pack-years of smoking, current smoking status, 6-min walk distance, body-mass index (BMI), forced expiratory volume in 1 s (FEV_1_), and modified medical council research (MMRC) dyspnea score, as available. See Table S1 for a listing of which covariates were available in each cohort. We performed stratified analyses based on smoking status and COPD case–control status (cases: GOLD 2–4, controls: normal spirometry).

We assessed predictive performance using area-under-the-receiver-operating-characteristic-curve (AUC) metrics, implemented in the pROC R package^22^. We tested the performance of the full multivariable models (age, sex, pack-years of smoking, current smoking status, 6-min walk distance, BMI, FEV_1_, MMRC) as well as a reduced clinical model (age, sex, race, pack-years of smoking) that is more reflective of information commonly available to primary care physicians. We examined single protein associations with time-to-death in univariable models and further stratified by smoking status. We also tested single protein associations with time-to-death in multivariable models, as described above. We examined the effect sizes of the protRS and single protein associations across cohorts using inverse variance fixed and random effects meta-analysis and forest plot visualizations using the meta R package^23^.

Respiratory- and cardiovascular-specific mortality outcomes are available in COPDGene (see Supplement for details on cause-of-death adjudication). We examined the performance of the protRS in the COPDGene testing set using multivariable Cox model regression models, as described above. To examine the relationship between the protRS and a previously described cardiovascular mortality score by Ganz et al.^16^, we calculated Pearson correlation coefficients between each protRS protein and Ganz score protein. We also performed linear regression between the Ganz score and the protRS and used the residuals as a risk score to examined the association of this protein risk score (Ganz residuals) with all-cause, respiratory-specific, and cardiovascular-specific mortality in the COPDGene testing set.

#### Biological characterization

To understand the biological effects of the protRS proteins, we used the protRS proteins as inputs into STRING (www.string-db.org) to construct a protein–protein interaction (PPI) network (5 interactors first shell, 5 interactors second shell), and performed MCL clustering (inflation factor 3) to identify modules associated with specific biological pathways. We also performed Reactome^25^ pathway enrichment on this PPI network. We performed Enrichr^26–28^ drug repurposing analyses to identify molecules that could reverse gene sets enriched in the protRS referencing the Multi-marker Analysis of GenoMic Annotation (MAGMA) Drug and Disease database^29^.

All analyses were performed in R v4.0.3. Normality was assessed by visual inspection of histograms. Univariable comparisons were performed with Student t-tests and categorical comparisons by analysis of variance (ANOVA). P-values less than 0.05 were considered nominally significant and values below a Bonferroni-corrected alpha were considered significant.

## Results

### Characteristics of study population

We included 2232 participants from three cohorts of smokers (COPDGene, LSC, SPIROMICS) and one general population cohort (MESA). Table 1 shows study participant characteristics. As expected, COPDGene, LSC, and SPIROMICS had lower mean baseline spirometry, and greater smoking exposure compared to the MESA general population cohort. LSC had the greatest proportion of females. COPDGene had the lowest proportion of African Americans. The COPDGene training and testing samples had similar characteristics.

**Table 1:** Characteristics of study participants.

n	COPDGene Training set	COPDGene Testing set	LSC	MESA	SPIROMICS
	543	550	217	683	239
Age in years (mean (SD))	62.17 (9.21)	61.65 (9.34)	55.63 (8.65)	68.82 (9.38)	61.18 (8.80)
Sex (No. % female)	285 (52.5)	278 (50.5)	171 (78.8)	351 (51.4)	108 (45.2)
Race					
African American	59 (10.9)	56 (10.2)	1(0.46)	133 (19.5)	55 (23.0)
non-Hispanic white	484 (89.1)	494 (89.8)	144(66.36)	275 (40.3)	171 (71.5)
East Asian	0	0	0(0)	53 (7.8)	0
Hispanic/LatinX	0	0	64(29.49)	222 (32.5)	0
Other	NA	NA	8(3.69)	NA	13 (5.4)
Body-mass index (Kg/m^2) (mean (SD))	28.73 (5.92)	28.67 (6.20)	27.34 (5.50)	29.01 (5.49)	28.08 (5.07)
Current smoking status (No. %)	208 (38.3)	200 (36.4)	132 (60.8)	57 (8.3)	96 (40.2)
Ever smoking status (No. %)	NA	NA	NA	397 (58.1)	NA
Pack-years of smoking (mean (SD))	43.49 (23.19)	46.19 (25.62)	41.23 (19.69)	10.40 (18.41)	46.79 (26.5)
FEV1% predicted (mean (SD))	77.56 (26.00)	77.33 (26.01)	88.10 (19.03)	95.32 (18.99)	82.82 (24.47)
FEV1/FVC ratio (mean (SD))	0.65 (0.17)	0.66 (0.17)	0.83 (0.17)	0.74 (0.09)	0.65 (0.15)
Dead (No. %)	70 (12.9)	73 (13.3)	47 (21.7)	93 (13.6)	48 (20.1)
Days followed (median [IQR])	2850.00 [2419.50, 3114.00]	2868.00 [2492.25, 3150.00]	6574.5[5478.8, 6939.8]	3184.00 [3020.50, 3319.00]	2906.00 [2270.50, 3353.50]

COPDGene, Genetic epidemiology of COPD study. MESA, Multi-Ethnic Study of Atherosclerosis. SPIROMICS, SubPopulations and InteRmediate Outcome Measures In COPD study. LSC, Lovelace Smokers’ Cohort. FEV1, forced expiratory volume in 1 s. FVC, forced vital capacity.

### Development of protein risk score

In Table S2, we show the associations of four proteomic models with time-to-death in the COPDGene testing sample. Of the tested models, the protein risk score (protRS) LASSO demonstrated the greatest hazard ratio (adj. HR 2.7 [95% CI 1.9–3.7], *p* = 3.0E−09) and was the most parsimonious model (i.e., included the smallest number of proteins). Based on cross-validation, the optimal number of proteins for this model was 15 with a lambda of 0.0463 (Figure S1). The weights and protein names are shown in Table S3 and a representative histogram of the rank-normalized protRS is shown in Figure S2.

To determine whether the protRS derived from SomaScan 1.3 K data was transferable to the 5 K platform, we calculated the protRS in a subset of COPDGene participants with 1.3 K proteomic data at baseline and 5 K proteomic data at the 5-year follow up visit (n = 660). We observed a high correlation between the scores (r = 0.7, Figure S3), even though BMPER was missing in the SomaScan 5 K dataset. The SomaScan 5 K protRS was associated with time-to-death in multivariable regression analysis (*p* = 0.0009). Based on these results, we carried the LASSO model trained on SomaScan 1.3 K data forward as the protRS for replication.

### A protein risk score predicts mortality

**Fig. 2:**
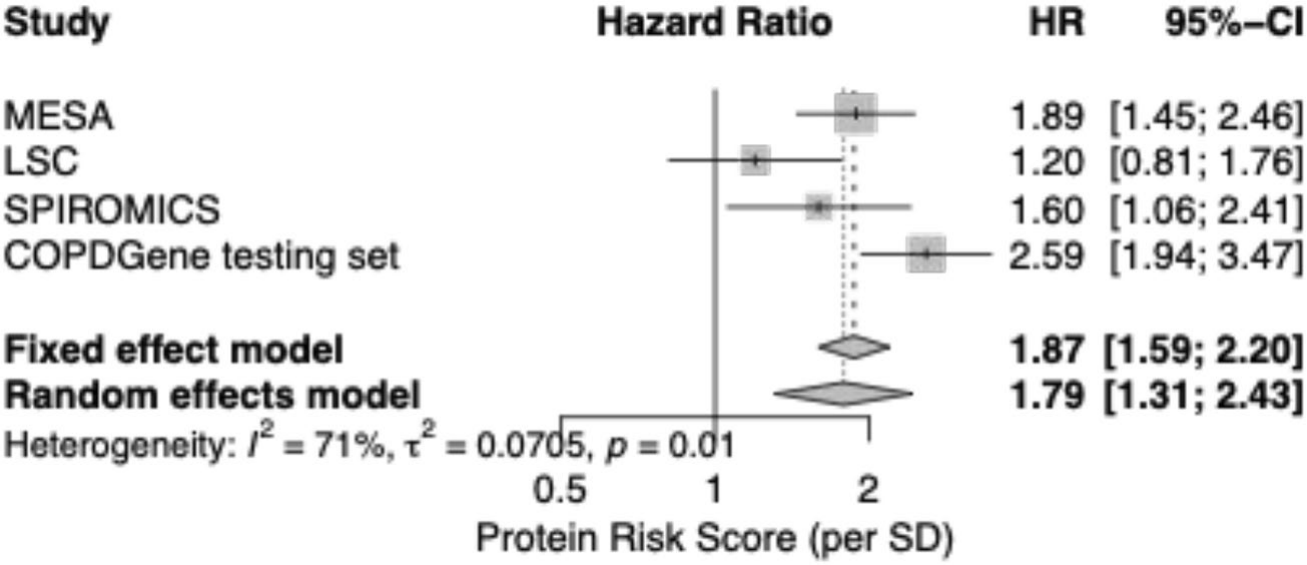
Forest plot demonstrating the association of the protein risk score (ProtRS) with all-cause mortality in testing cohorts. See Table 1 for abbreviations.

**Fig. 3:**
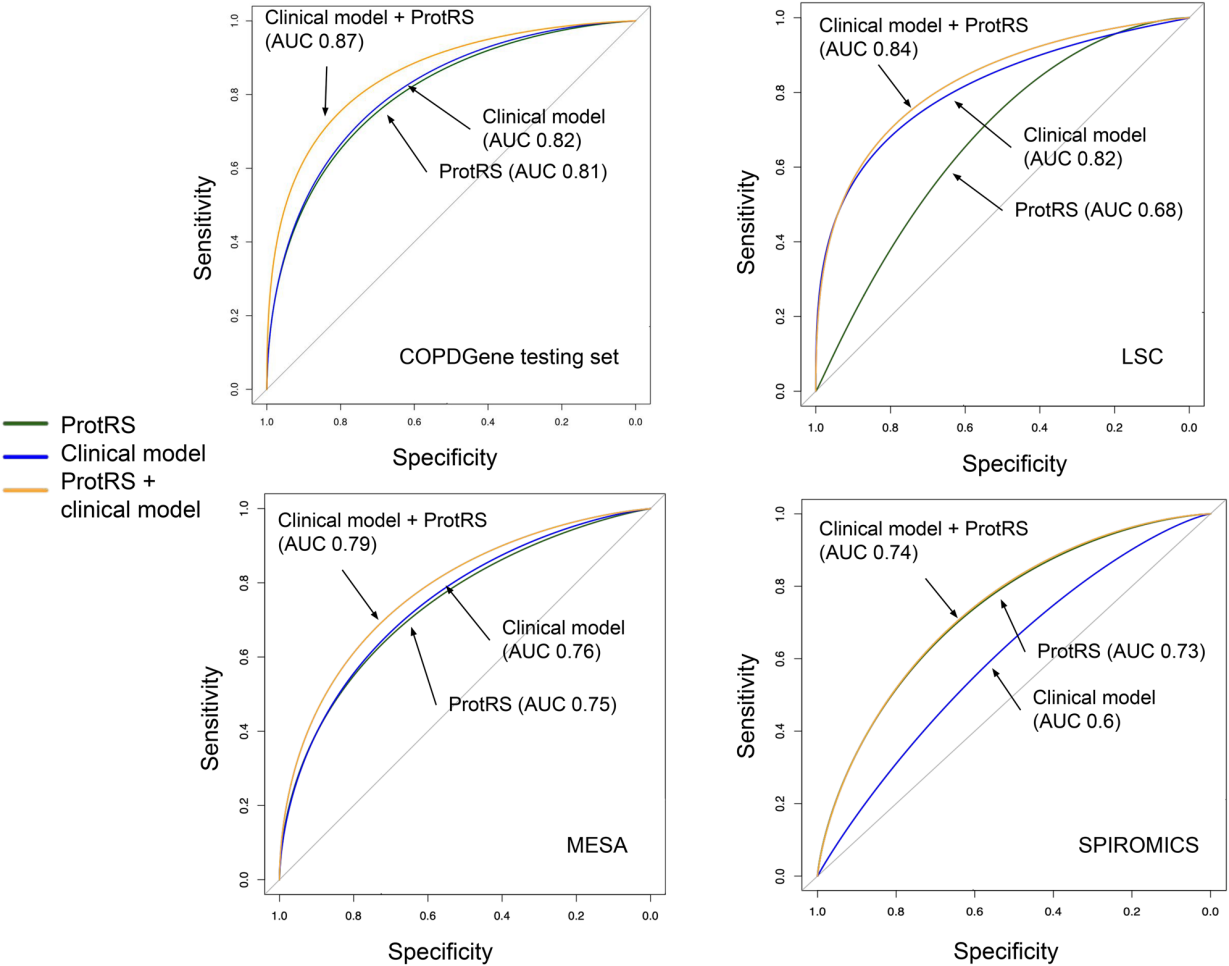
Receiver-operating-characteristic-curve (ROC) and area-under-the-ROC-curve (AUC) analysis in each cohort. ProtRS, protein risk score. Clinical model includes the reduced clinical model with age, sex, race, and smoking variables (pack-years or ever smoking status, depending on cohort).

The univariable association of the protRS with mortality in each cohort is shown in Figure S4. In meta-analysis of multivariable models, the protRS was associated with all-cause-mortality (random-effects HR 1.79 [95% CI 1.31–2.43]; Fig. 2 and Table 2). We observed significant cohort heterogeneity (I^2^ = 71.5%). In modified D’Agostino-Nam tests, only the LSC model was significantly mis-calibrated (*p* < 0.05). In stratified analyses, the observed associations are of similar effect sizes across strata except for current and former smokers in LSC and COPD cases in SPIROMICS (Table 2). In AUC analyses, adding the protRS to a simple clinical model (age, sex, race, and pack-years of smoking) improved all-cause mortality prediction in the COPDGene testing sample and SPIROMICS, with a trend toward improved predictive performance in LSC and MESA (Table S4, Fig. 3). Combining the protRS with the full clinical model, which included BODE variables, significantly improved performance the COPDGene testing sample but not in other cohorts.

**Table 2:** Adjusted hazard ratios for the protRS in the overall cohorts and stratified analyses in the COPDGene testing set, LSC, MESA, and SPIROMICS.

*Stratum*	COPDGene testing set (n = 550)		LSC (n-217)		MESA (n = 683)		SPIROMICS (n = 239)	
	HR (95% CI)	*p*	HR (95% CI)	*p*	HR (95% CI)	*p*	HR (95% CI)	*p*
Current smokers	2.3 (1.3–4.1)	0.0057	1.3 (0.74–2.3)	0.35	3.4 (0.8–15)	0.098	2 (1–3.7)	0.041
Former smokers	2.8 (1.8–4.1)	8.8E−07*	0.94 (0.58–1.6)	0.82	1.7 (1.3–2.3)	0.00011*	1.6 (0.83–3.3)	0.16
Ever smokers	2.6 (1.9–3.5)	1.60E−10*	1.1 (0.66–1.9)	0.68	2 (1.4–2.7)	8.20E-05*	1.6 (1.1–2.4)	0.026
Never smokers	NA	NA	1.3 (0.7–2.5)	0.39	1.7 (1.1–2.7)	0.013	NA	NA
COPD cases (GOLD 2–4)	2.7 (1.8–4.1)	1.70E−06*	NA	NA	1.7 (0.79–3.6)	0.17	1.2 (0.65–2.2)	0.57
Controls (GOLD 0)	2.7 (1.3–5.6)	0.0099	NA	NA	1.8 (1.2–2.5)	0.0023*	4.6 (1.8–12)	0.0016*
Overall	2.6 (1.9–3.5)	1.60E–10*	1.2 (0.81–1.8)	0.36	1.9 (1.4–2.5)	2.5E–06*	1.6 (1.1–2.4)	0.026

Multivariable models were adjusted for age, sex, self-reported race, current smoking status, pack years of smoking (when available), FEV_1_% predicted, BMI, MMRC dyspnea score, and 6-min walk distance. GOLD = Global Initiative for Chronic Obstructive Lung Diseases. HR, hazard ratio. See Table 1 legend for other abbreviations. *, below Bonferroni threshold of 0.05/4 cohorts/5 strata = 0.0025. All included COPDGene and SPIROMICS participants are ever smokers.

Having demonstrated the association of the protRS with mortality in multiple cohorts, we sought to understand how individual proteins within the risk score are associated with mortality. The adjusted hazard ratios for individual risk score proteins in each cohort and meta-analyses are shown in Table S5. Corresponding forest plots are shown in Figure S5. The proteins with the least effects size heterogeneity (i.e., lowest I^2^) across cohorts were SOD1, GHR, CXCL13, CSF1, and GDF15. We observed that only 5 of the 15 proteins demonstrated consistent directions of effect across cohorts (TFF3, GDF15, CXCL13, CXCL8, GHR).

A subset of COPDGene testing sample individuals had cause-specific mortality data, and the protRS demonstrated a greater association with respiratory compared to cardiovascular mortality (Table S6). To better understand the protRS association with respiratory mortality, we compared our score with the Ganz cardiovascular (CV) mortality score (hereafter, the “Ganz score”)^16^. Two proteins were in common between our protRS and the Ganz score, which had opposite directions of effects (C7: 0.73 in protRS, -2.12 in Ganz score; SERPINF2: -1.2 in protRS, 2.64 in Ganz score). A correlation matrix of Pearson correlation coefficients for the protRS and Ganz score proteins is shown in Figure S6; four proteins had average correlation coefficients ≥ 0.1 (SERPINF2, C7, GDF15, TNNT2). We observed that the protRS and the Ganz score were highly correlated, and that this relationship was attenuated after regressing out the Ganz score (Figure S7). Using the protRS (Ganz residuals), we observed that this score had a smaller effect on all-cause and cardiovascular-specific mortality and a larger effect on respiratory-specific mortality (Table S7).

### Biological characterization of proteins

As LASSO optimizes feature selection for the purposes of prediction, the selected features are not necessarily causally related to the outcome. Therefore, we mapped protRS proteins to the protein–protein interactome to construct a PPI network (Figure S8) and performed Reactome pathway enrichment (Table S8) and MCL clustering (Table S9) analyses to gain insights into the biological processes captured by the protRS. Enrichment analyses suggest that alterations in complement activation, innate immunity, cytokine signaling (e.g., IL-10), Wnt signaling, and RUNX1 activation are important determinants of mortality in smokers. Network-based clustering analysis identified four clusters that suggest a combination of cytokine signaling, cardiovascular mortality factors, and innate immune dysfunction may play a role in mortality in smokers (see Figure S8 legend and Table S9 for cluster details). In drug repurposing analyses, we found that the protRS was enriched for gene sets reversed by pamidronate, glucocorticoid receptor antagonists, PDGFR inhibitors, VEGF inhibitors, macrolide antibiotics, and proton-pump inhibitors, amongst others (Table S10).

## Discussion

In this study of over 2000 participants from smoking and general population cohorts, we demonstrated that a 15-protein risk score (protRS) was associated with time-to-death (all-cause mortality) and in certain populations of smokers can improve prediction compared to a set of commonly available clinical predictors. These proteins appear to be related to both cardiovascular and respiratory mortality, with a greater effect on respiratory mortality. We identified chemo- and cyto-kine signaling, TNF signaling, responses to infections and activation of innate immunity, extracellular matrix turnover, and growth hormone signaling as possible drivers of mortality in smokers. Drug repurposing analyses suggest that several existing agents (e.g., pamidronate, macrolide antibiotics, proton-pump inhibitors) could be beneficial to the subset of individuals with an elevated protRS.

While the protRS demonstrated strong associations with all-cause mortality, the improvement in prediction over age, sex, smoking, and other factors was variable. There was no significant improvement in LSC or MESA, and it only improved predictive capacity, as measured by AUC, over BODE^8^ variables in COPDGene and over a reduced set of clinical risk factors in COPDGene and SPIROMICS. Amongst the testing cohorts, COPDGene and SPIROMICS are the most similar, and as the protRS was derived in COPDGene, it appears to be most applicable to older smokers that are primarily non-Hispanic white and African American. By contrast, MESA is a general population cohort and LSC recruited more LatinX and female (79%) participants than other cohorts. An important caveat is that the clinical variables were not predicted values but were the actual variables from each cohort, which means the clinical variable estimates are likely overfitted. Indeed, the performance characteristics of these clinical variables within individual cohorts are much higher than reported in the literature^8,11^, alluding to the issue of overfitting—and given that these are subsets of individuals with proteomic data—there could be selection bias for which we are not able to account. As is, the clinical utility of the protRS is likely limited to individuals who would meet inclusion criteria for COPDGene or SPIROMICS. While we advocate for measurement of the most important predictors of mortality (6-min walk distance^11,12^ and FEV_1_^8^), we acknowledge the challenges of obtaining these measures in a primary care setting. The role of blood-based biomarkers, such as the one presented here, could also aid in early referral to pulmonary specialists.

Proteomics have been successful in identifying predictors of all-cause and cardiovascular-specific mortality. However, the protRS demonstrated greater association with respiratory-specific mortality compared to other tested models. We systematically compared the protRS to a previously published cardiovascular mortality score^16^ (i.e., Ganz score) and found that, after regressing out the Ganz score, the protRS had a larger effect on respiratory-specific compared to all-cause or cardiovascular-specific mortality. The most highly correlated proteins were SERPINF2, C7, GDF15, and TNNT2. SERPINF2 and C7 are in the Ganz score, albeit with opposite directions of effects; the opposite effect directions may represent smoking effects or noise in the proteomic dataset. TNNT2 is the gene that encodes Troponin-T, which is found only in heart muscle and is used clinically as a marker of cardiac ischemia. GDF15 has been identified in multiple proteomic analyses related to mortality and we observed that it interacts with TNNT2 in our network analysis. These four protRS proteins are likely driving much of the observed associations with cardiovascular mortality.

Amongst the remaining proteins, five (GHR, CXCL13, TFF3, CXCL8, TNFSF1) were significantly associated with mortality in meta-analysis, which suggests that these proteins are likely related to respiratory mortality in smokers. While LASSO provides automated feature selection and minimizes collinearity, the selected features are not necessarily causal^30^—rather, the selected proteins (i.e., features) might interact with causal proteins. For these reasons, we mapped protRS proteins to the human interactome and constructed a protein–protein interaction (PPI) network. We identified four large clusters of proteins. Of interest, one cluster was a chemokine/cytokine cluster that linked to TNFSF15 and another cluster suggested that SERPINF2 provides a link to complement activation. Tumor necrosis factors (TNFs) are involved in regulation of growth, airway hyperresponsiveness, inflammation, and immunomodulation^31,32^. TNF-α levels are elevated in COPD patients compared to controls^33^ and TNF signaling has been implicated in several pulmonary diseases, including COPD^31^. TNF antagonists have demonstrated promise in observational studies^34^ and demonstrated similar efficacy as prednisone for reducing COPD exacerbations in a clinical trial yet was less effective in the subgroup with eosinophilia^35^. We identified pentoxyfylline as a drug repurposing candidate, but the clinical utility is limited by adverse effects and drug-drug interactions. Perhaps downstream TNF pathway targets or other agents warrant further investigation. While we used a parsimonious set of risk score proteins for drug repurposing analyses, a more comprehensive set of proteomic drivers of respiratory-specific mortality could provide a more ideal set of proteins on which to base such analyses. Future studies using alternate feature selection methods could quantify the set of proteins that explain the majority of respiratory-specific mortality and enhance identification of drug repurposing agents. Complement activation has been observed to rise with COPD exacerbations (one of the major drivers of COPD mortality) and to be positively correlated to CRP levels^36^. Taken together, the protRS may identify individuals at high risk of mortality for which TNF, chemokine, and complement pathways may be potential targets.

Strengths of this study include that we demonstrate our findings in multiple cohorts, both smoking and general population, and across both SomaScan 1.3 K and 5 K platforms. While we advocate for measurement of spirometry and 6-min walk distance in COPD patients, the protRS may provide a practical blood-based alternative for predicting mortality in heavy smokers in the primary care setting. Further, we examined protRS proteins in the context of the human protein–protein interactome and identified likely molecular drivers of respiratory mortality which might be targeted by existing compounds.

One limitation is that the protRS appears most applicable to heavy smokers recruited from a predominantly non-Hispanic white and African American United States population. We were not able to test the performance of the protRS in real-world cohorts, and ultimately, a prospective trial would be needed to truly validate any biomarker of COPD mortality. Given the limited sample sizes across cohorts, the number of deaths was relatively small, though we still demonstrate power to detect an association between the protRS and mortality. Observational studies of mortality can be susceptible to immortal time bias, but we did not measure mortality prior to the start of the study or proteomic measurements, so there was not a pre-exposure time period in which an event could occur—that is, the time at which we started measuring survival is the same as the time we collected blood samples. For clinical application, additional research would be needed to understand when to measure the protRS, who is considered at ‘high risk’, and what potential therapies should be tested in a ‘high risk’ subgroup.

In conclusion, a blood-based protein risk score predicted mortality in heavy smokers and was complementary to commonly used clinical risk factors. This risk score includes proteins that implicate signaling pathways related to both respiratory and cardiovascular mortality.

## Supplementary Information

Supplementary Information.

## Data Availability

NHLBI TOPMed Whole Genome Sequencing (phs001607) and proteomic data (phs001416.v3.p1) are available through the database of Genotype and Phenotypes (dbGaP).
