# Supplementary material for: A protein risk score for all-cause and respiratory-specific mortality in non-Hispanic white and African American individuals who smoke
Source: Sci Rep. 2024 Sep 4;14:20618. doi: 10.1038/s41598-024-71714-7 (PMC11374806; doi:10.1038/s41598-024-71714-7)
Supplement: Supplementary file 1 — Supplementary Information. [file 41598_2024_71714_MOESM1_ESM.docx]

**Supplement to “A Protein Risk Score for All-Cause and Respiratory-specific Mortality in non-Hispanic White and African American Individuals Who Smoke”**

[Funding and Acknowledgements 2](#_Toc162375502)

[Supplementary Methods 6](#_Toc162375503)

[Additional cohort details 6](#_Toc162375504)

[COPDGene 6](#_Toc162375505)

[SPIROMICS 7](#_Toc162375506)

[LSC 8](#_Toc162375507)

[MESA 8](#_Toc162375508)

[Supplementary Results 10](#_Toc162375509)

[Supplementary Figures 10](#_Toc162375510)

[Supplementary Tables 26](#_Toc162375511)

[References 42](#_Toc162375512)

## Funding and Acknowledgements

**COPDGene Phase 3 Grant Support and Disclaimer**

The project described was supported by Award Number U01 HL089897 and Award Number U01 HL089856 from the National Heart, Lung, and Blood Institute. The content is solely the responsibility of the authors and does not necessarily represent the official views of the National Heart, Lung, and Blood Institute or the National Institutes of Health.

**COPD Foundation Funding**

COPDGene is also supported by the COPD Foundation through contributions made to an Industry Advisory Board that has included AstraZeneca, Bayer Pharmaceuticals, Boehringer- Ingelheim, Genentech, GlaxoSmithKline, Novartis, Pfizer, and Sunovion.

**COPDGene® Investigators – Core Units**

*Administrative Center*: James D. Crapo, MD (PI); Edwin K. Silverman, MD, PhD (PI); Barry J. Make, MD; Elizabeth A. Regan, MD, PhD

*Genetic Analysis Center*: Terri H. Beaty, PhD; Peter J. Castaldi, MD, MSc; Michael H. Cho, MD, MPH; Dawn L. DeMeo, MD, MPH; Adel El Boueiz, MD, MMSc; Marilyn G. Foreman, MD, MS; Auyon Ghosh, MD; Lystra P. Hayden, MD, MMSc; Craig P. Hersh, MD, MPH; Jacqueline Hetmanski, MS; Brian D. Hobbs, MD, MMSc; John E. Hokanson, MPH, PhD; Wonji Kim, PhD; Nan Laird, PhD; Christoph Lange, PhD; Sharon M. Lutz, PhD; Merry-Lynn McDonald, PhD; Dmitry Prokopenko, PhD; Matthew Moll, MD, MPH; Jarrett Morrow, PhD; Dandi Qiao, PhD; Elizabeth A. Regan, MD, PhD; Aabida Saferali, PhD; Phuwanat Sakornsakolpat, MD; Edwin K. Silverman, MD, PhD; Emily S. Wan, MD; Jeong Yun, MD, MPH

*Imaging Center*: Juan Pablo Centeno; Jean-Paul Charbonnier, PhD; Harvey O. Coxson, PhD; Craig J. Galban, PhD; MeiLan K. Han, MD, MS; Eric A. Hoffman, Stephen Humphries, PhD; Francine L. Jacobson, MD, MPH; Philip F. Judy, PhD; Ella A. Kazerooni, MD; Alex Kluiber; David A. Lynch, MB; Pietro Nardelli, PhD; John D. Newell, Jr., MD; Aleena Notary; Andrea Oh, MD; Elizabeth A. Regan, MD, PhD; James C. Ross, PhD; Raul San Jose Estepar, PhD; Joyce Schroeder, MD; Jered Sieren; Berend C. Stoel, PhD; Juerg Tschirren, PhD; Edwin Van Beek, MD, PhD; Bram van Ginneken, PhD; Eva van Rikxoort, PhD; Gonzalo Vegas Sanchez- Ferrero, PhD; Lucas Veitel; George R. Washko, MD; Carla G. Wilson, MS;

*PFT QA Center, Salt Lake City, UT*: Robert Jensen, PhD

*Data Coordinating Center and Biostatistics*, *National Jewish Health, Denver, CO*: Douglas Everett, PhD; Jim Crooks, PhD; Katherine Pratte, PhD; Matt Strand, PhD; Carla G. Wilson, MS

*Epidemiology Core*, *University of Colorado Anschutz Medical Campus, Aurora, CO*: John E. Hokanson, MPH, PhD; Erin Austin, PhD; Gregory Kinney, MPH, PhD; Sharon M. Lutz, PhD; Kendra A. Young, PhD

*Mortality Adjudication Core:* Surya P. Bhatt, MD; Jessica Bon, MD; Alejandro A. Diaz, MD, MPH; MeiLan K. Han, MD, MS; Barry Make, MD; Susan Murray, ScD; Elizabeth Regan, MD; Xavier Soler, MD; Carla G. Wilson, MS

*Biomarker Core*: Russell P. Bowler, MD, PhD; Katerina Kechris, PhD; Farnoush Banaei- Kashani, PhD

**COPDGene® Investigators – Clinical Centers**

*Ann Arbor VA:* Jeffrey L. Curtis, MD; Perry G. Pernicano, MD

*Baylor College of Medicine, Houston, TX*: Nicola Hanania, MD, MS; Mustafa Atik, MD; Aladin Boriek, PhD; Kalpatha Guntupalli, MD; Elizabeth Guy, MD; Amit Parulekar, MD;

*Brigham and Women’s Hospital, Boston, MA*: Dawn L. DeMeo, MD, MPH; Craig Hersh, MD, MPH; Francine L. Jacobson, MD, MPH; George Washko, MD

*Columbia University, New York, NY*: R. Graham Barr, MD, DrPH; John Austin, MD; Belinda D’Souza, MD; Byron Thomashow, MD

*Duke University Medical Center, Durham, NC*: Neil MacIntyre, Jr., MD; H. Page McAdams, MD; Lacey Washington, MD

*HealthPartners Research Institute, Minneapolis, MN*: Charlene McEvoy, MD, MPH; Joseph Tashjian, MD

*Johns Hopkins University, Baltimore, MD*: Robert Wise, MD; Robert Brown, MD; Nadia N. Hansel, MD, MPH; Karen Horton, MD; Allison Lambert, MD, MHS; Nirupama Putcha, MD, MHS

*Lundquist Institute for Biomedical Innovation at Harbor UCLA Medical Center, Torrance, CA*: Richard Casaburi, PhD, MD; Alessandra Adami, PhD; Matthew Budoff, MD; Hans Fischer, MD; Janos Porszasz, MD, PhD; Harry Rossiter, PhD; William Stringer, MD

*Michael E. DeBakey VAMC, Houston*, *TX*: Amir Sharafkhaneh, MD, PhD; Charlie Lan, DO *Minneapolis VA:* Christine Wendt, MD; Brian Bell, MD; Ken M. Kunisaki, MD, MS

*Morehouse School of Medicine, Atlanta, GA*: Eric L. Flenaugh, MD; Hirut Gebrekristos, PhD; Mario Ponce, MD; Silanath Terpenning, MD; Gloria Westney, MD, MS

*National Jewish Health, Denver, CO*: Russell Bowler, MD, PhD; David A. Lynch, MB *Reliant Medical Group, Worcester, MA*: Richard Rosiello, MD; David Pace, MD

*Temple University, Philadelphia, PA:* Gerard Criner, MD; David Ciccolella, MD; Francis Cordova, MD; Chandra Dass, MD; Gilbert D’Alonzo, DO; Parag Desai, MD; Michael Jacobs, PharmD; Steven Kelsen, MD, PhD; Victor Kim, MD; A. James Mamary, MD; Nathaniel Marchetti, DO; Aditi Satti, MD; Kartik Shenoy, MD; Robert M. Steiner, MD; Alex Swift, MD; Irene Swift, MD; Maria Elena Vega-Sanchez, MD

*University of Alabama, Birmingham, AL:* Mark Dransfield, MD; William Bailey, MD; Surya P. Bhatt, MD; Anand Iyer, MD; Hrudaya Nath, MD; J. Michael Wells, MD

*University of California, San Diego, CA*: Douglas Conrad, MD; Xavier Soler, MD, PhD; Andrew Yen, MD

*University of Iowa, Iowa City, IA*: Alejandro P. Comellas, MD; Karin F. Hoth, PhD; John Newell, Jr., MD; Brad Thompson, MD

*University of Michigan, Ann Arbor, MI:* MeiLan K. Han, MD MS; Ella Kazerooni, MD MS; Wassim Labaki, MD MS; Craig Galban, PhD; Dharshan Vummidi, MD

*University of Minnesota, Minneapolis, MN*: Joanne Billings, MD; Abbie Begnaud, MD; Tadashi Allen, MD

*University of Pittsburgh, Pittsburgh, PA*: Frank Sciurba, MD; Jessica Bon, MD; Divay Chandra, MD, MSc; Joel Weissfeld, MD, MPH

*University of Texas Health, San Antonio, San Antonio, TX*: Antonio Anzueto, MD; Sandra Adams, MD; Diego Maselli-Caceres, MD; Mario E. Ruiz, MD; Harjinder Singh

The **ECLIPSE** study (NCT00292552; GSK code SCO104960) was funded by GlaxoSmithKline.

The **Lovelace Smokers Cohort** was recruited through Lovelace Scientific Resources, Albuquerque, NM, USA under the direction of Darlene Harbour, by funding from the State of New Mexico appropriation from the Tobacco Settlement Fund.

## Supplementary Methods

### Additional cohort details

#### COPDGene

*SomaScan data:* We measured blood proteomic data using SOMAscan Human Plasma 1.3K (version 3.0), which uses aptamers (i.e. SOMAmers) to quantify 1,305 unique human proteins. Plate hybridization, median signal normalization, and plate scaling and calibration of SOMAmers were performed to control for variability across array signals, inter-run variability, inter-assay variation between analytes and batch differences between plates. Data were log-transformed prior to statistical analysis. Further details regarding preparation of SomaScan data has been previously published^1^. The study started as a case-control study and was extended to a longitudinal study with 10-year follow up mortality data. At the five-year follow up visit, blood samples on 5,670 individuals were collected and proteomic data measured using SomaScan 5K (version 4.0), processing the data in the same fashion with 2 additional normalization steps. Median normalization to a reference using adaptive normalization by maximum likelihood is applied within SOMAmer dilution group to quality control replicates and on individual samples to remove edge effect and technical variance^2^. The SomaScan 5K data were used to validate whether a protein risk score derived on SomaScan 1.3K data would be transferrable to 5K data.

*Mortality data:* Death was adjudicated by searching the social security death index (SSDI) database; a central search was performed on January 31, 2018, and deaths were back-censored three months to account for lag time between death and appearance in the SSDI database. Nine sites performed local SSDI searches at varying dates, and deaths were also back-censored three months.

*Cause of death adjudication:* For cause of death adjudication, six physicians reviewed death certificates, medical records, and informant interviews. Two reviewers classified each death as a single primary cause of death according to modified TORCH criteria^3^; 84% of deaths were adjudicated and classified as respiratory, cardiovascular, cancer, or other causes of death. For this study, we examined only respiratory and cardiovascular mortality.

#### SPIROMICS

*SomaScan data:* For validation we used the smokers (stratum 2-4) at visit 1 who have SomaScan data (n=239). Our study population is multiracial with 71.5% non-Hispanic white, 17.1% African American and 5.4% reporting other races. SomaScan 1.3K data was measured on fasting EDTA plasma from 288 subjects from Visit 1.

*Mortality data:* Mortality data used in this analysis was from the mortality dataset ‘DEV_Mortality_INV1_20221004’. It contains the status of the participant as of December 31, 2021. Days are from enrollment to death or withdraw, if withdrew before death, or last contact. All others were censored on December 31. 2021.

#### LSC

*Somacan data:* Samples from rapid decliners (defined as FEV1 loss ± 30 ml/year, n=217) and non-decliners, subjects with and without chronic bronchitis were randomly selected for assay on an expanded SomaScan Version 3 platform which included approximately 5,000 aptamers. Samples were from visit 1. Details regarding preparation of SomaScan data has been previously published^4^.

*Mortality data:* Two National Death Index (NDI) searches were completed in 2014 and mid 2020 and a total 380 deaths out of the 2372 subjects were identified (Table 1). Primary causes of death were coded using International Classification of Diseases-10 (ICD-10).

#### MESA

SomaScan data: Plasma protein levels were measured at baseline Exam 1 using the SOMAscan HTS Assay 1.3K. As part of the TOPMed MESA Multi-Omics project, 900 participants were selected for MESA Exam 1 proteomic profiling based on the following criteria: (1) restrict to those already included in the TOPMed Whole Genome Sequencing effort^5^, (2) preserve the race/ethnic distribution of participants in the parent MESA cohort, (3) maximize the amount of overlapping ‘omics data (with the other ‘omics included in the TOPMed MESA Multi-Omics pilot requiring availability of plasma samples for metabolomics, RNA from PBMCs; monocytes or T cells for RNA-seq, and whole blood for DNA methylation).

*Mortality data:* Participants were followed starting from the baseline examination with systematic ascertainment and adjudication of CVD events and mortality (adjudicated through 2017)^6^.

## Supplementary Results

## Supplementary Figures

Figures S1: Cross-validation plot generated by least absolute shrinkage and selection operator (LASSO) demonstrating the lambda (log lambda (*x-axis*)) at which the C-index (*y-axis*) is optimized. The top axis shows the number of features selected at each lambda value.


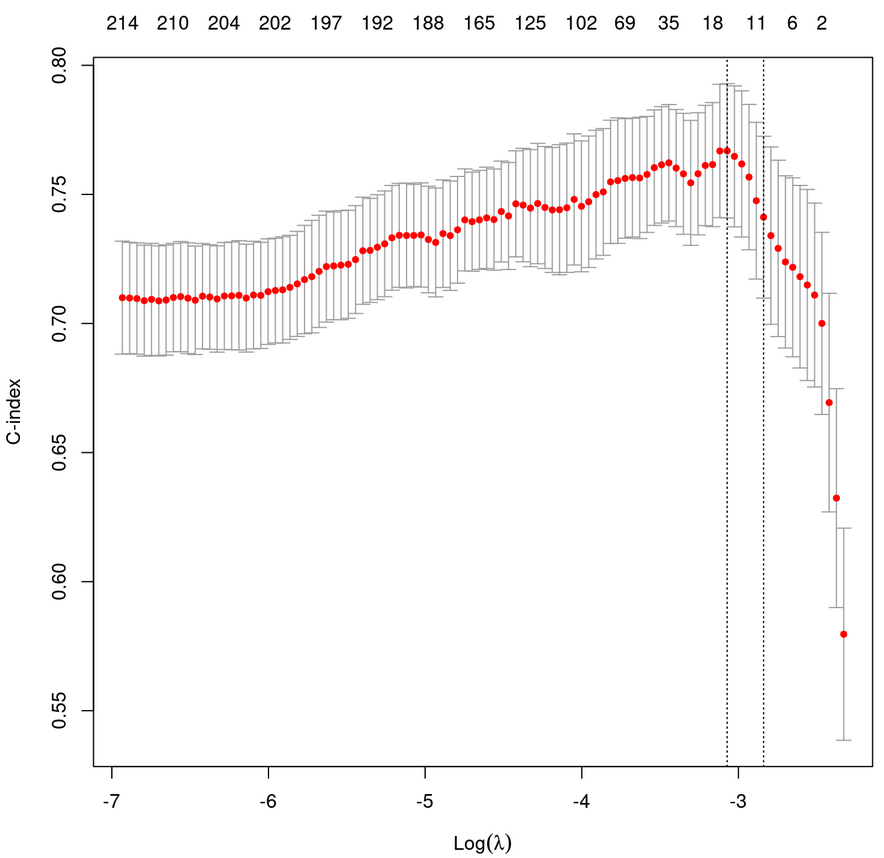


Figure S2: Representative histogram of protein risk score (protRS) in the COPDGene testing cohort. The protRS was rank normalized to facilitate statistical analysis.


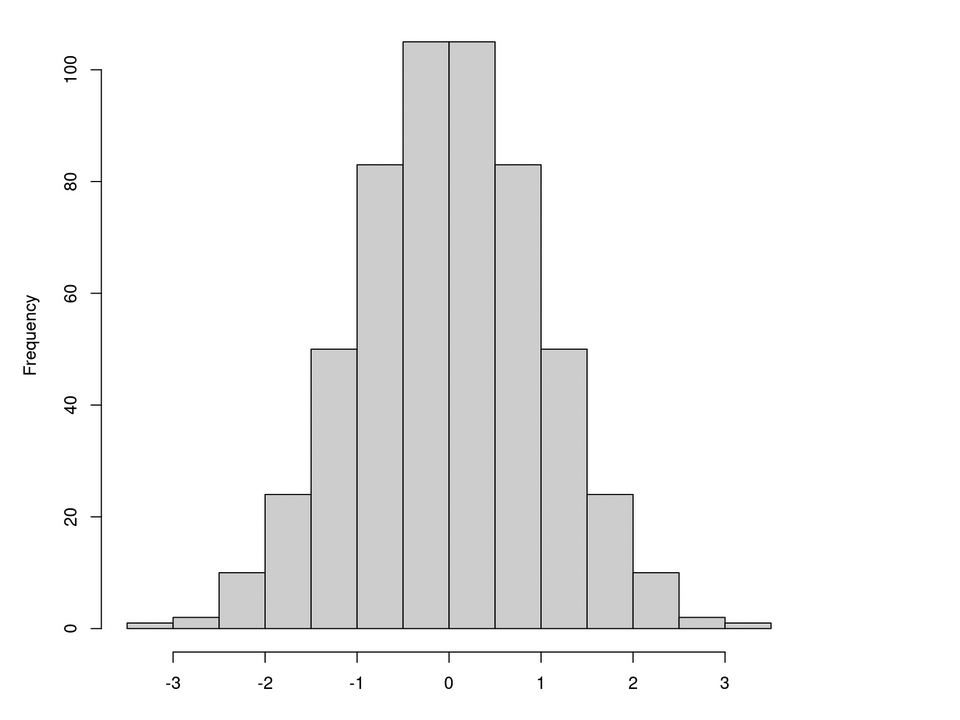


Figure S3. Protein risk scores (protRSs) derived from 1.3K and 5K Somascan data in a subset of patients with both assays.


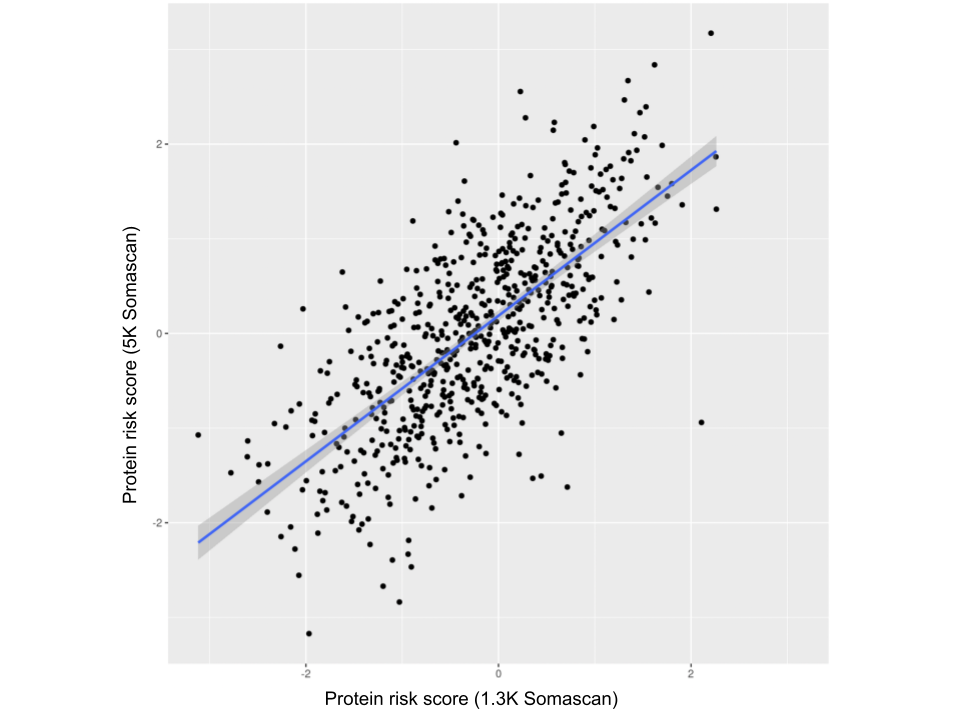


Figure S4: Boxplots showing the association of the protein risk score (protRS) with all-cause mortality in each cohort. *** p-value < 0.001. See Figure 1 legend for abbreviations.

Figure S5: Forest plots showing the association of each protein in the protein risk score in fixed and random effects inverse variance meta-analysis.


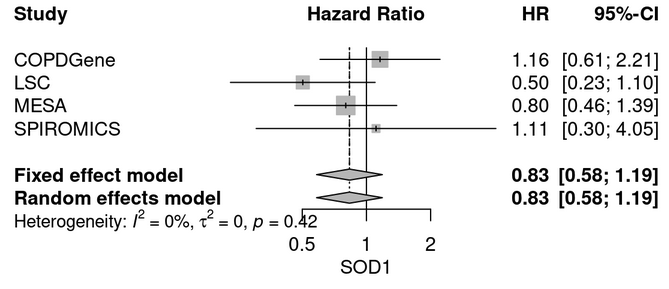


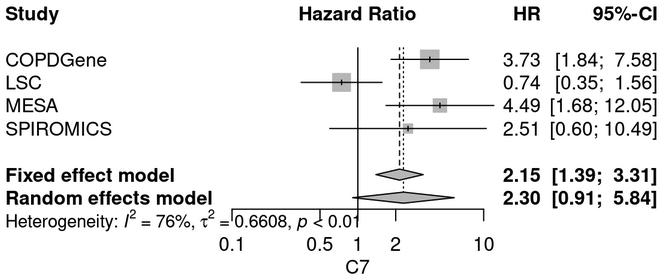


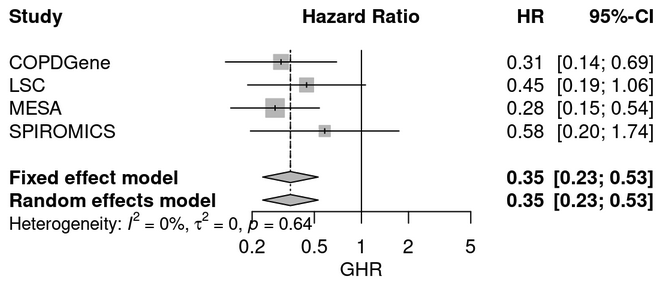


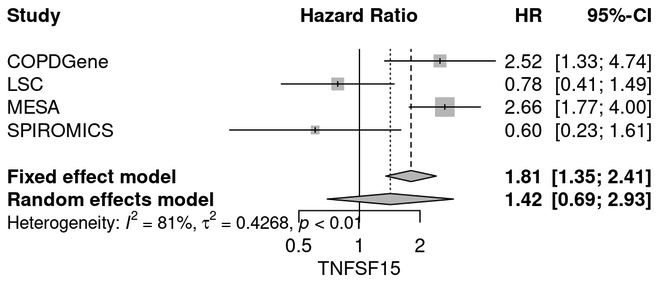


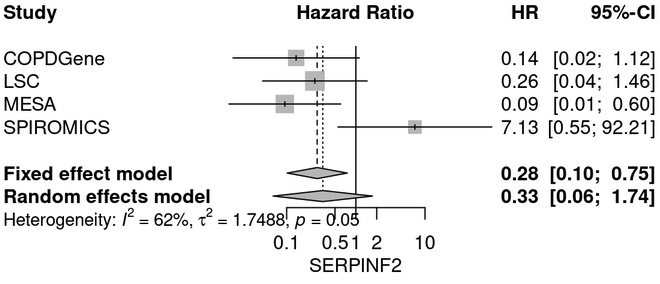


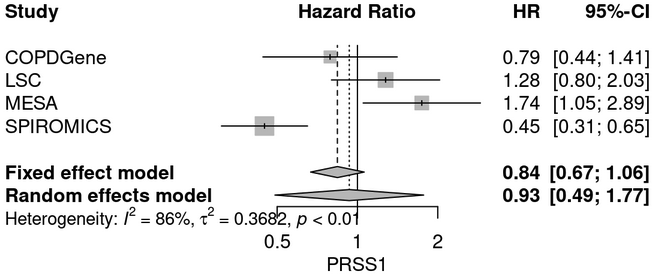


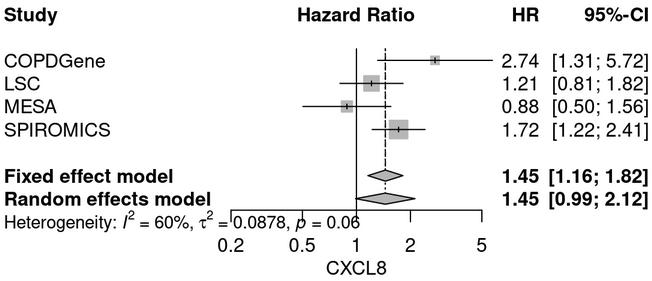


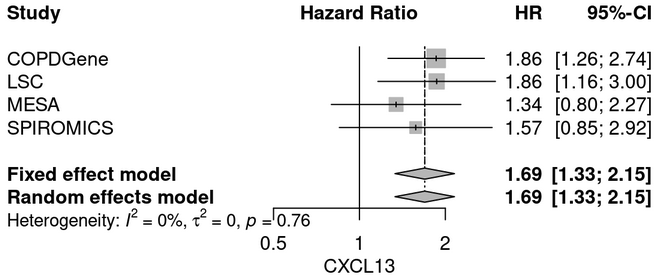


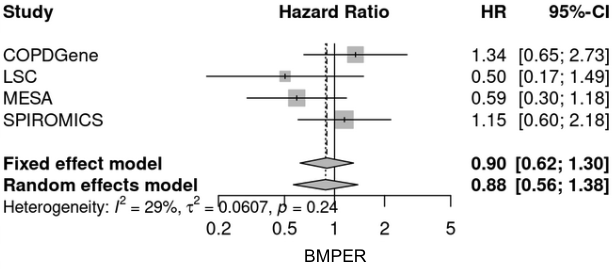


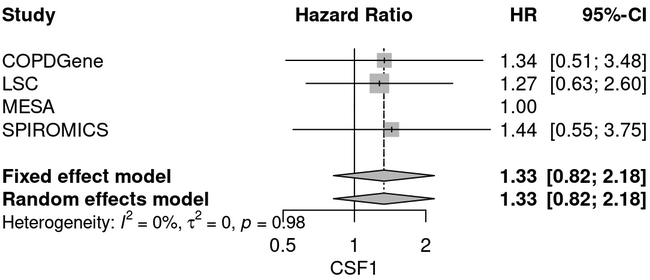


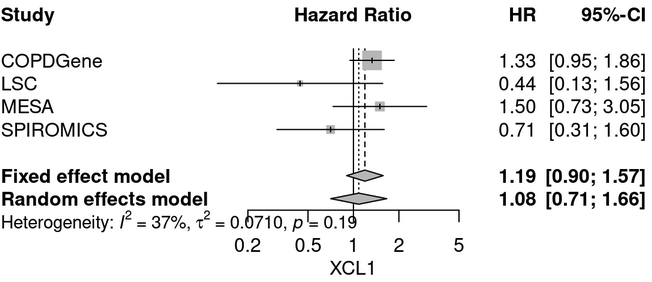


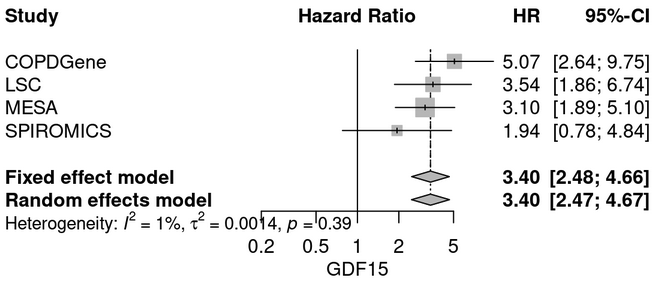


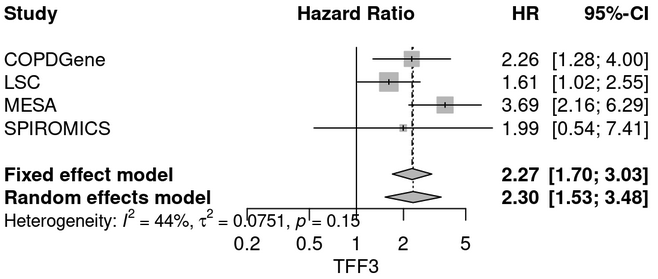


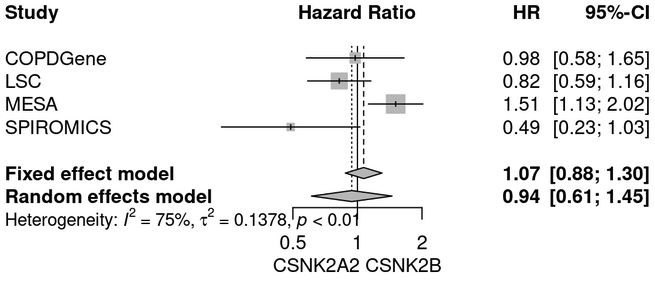


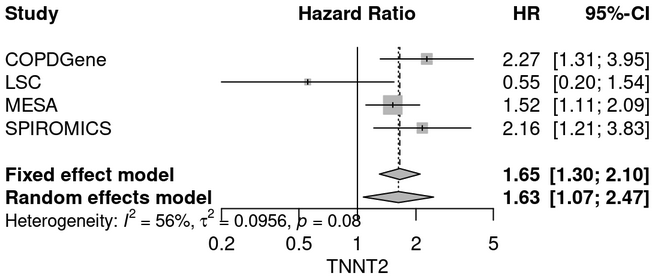


Figure S6: Pearson correlation coefficients for each protein in the ProtRS with each protein in the Ganz cardiovascular mortality score.


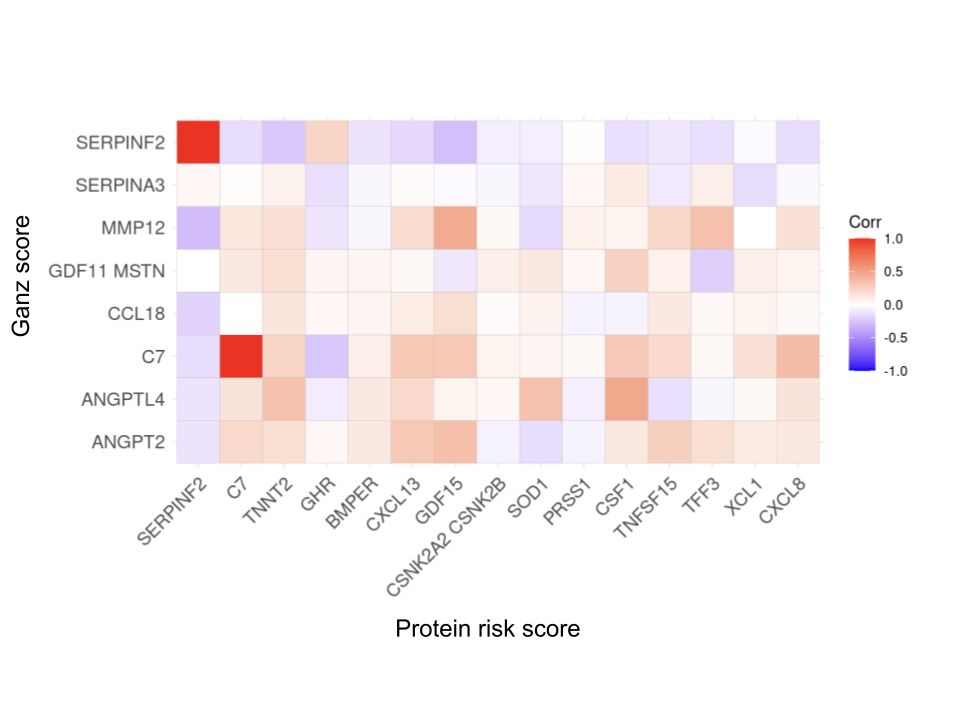


Figure S7: Scatterplots showing the relationship between the Ganz cardiovascular mortality score and the ProtRS before (A) and after (B) regressing out the Ganz score.


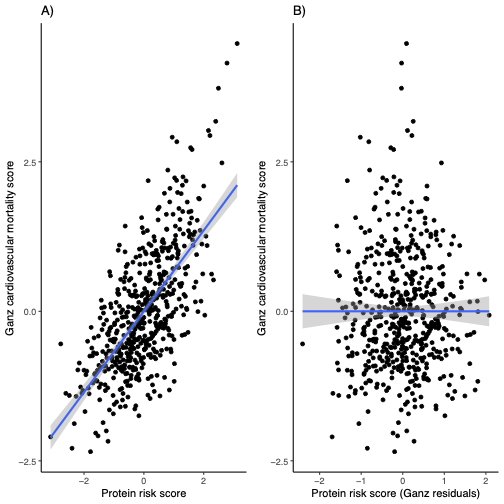


Figure S8: STRING network based on the 15 proteins in the protein risk score. Proteins were allowed up to 5 interactors in the first shell and 5 interactors in the second shell. MCL clustering was performed (inflation parameter 3) as indicated by the colors. The red cluster implicates CXCR and cytokine signaling as well as a link to TNFSF15. The light green cluster implicates SERPINF2 and activation of innate immunity through complement pathways. The dark green cluster implicates changes in protein phosphorylation states. The blue cluster visualizes the interaction of GDF15 with TNNT2, which is an enzyme in cardiac muscle.


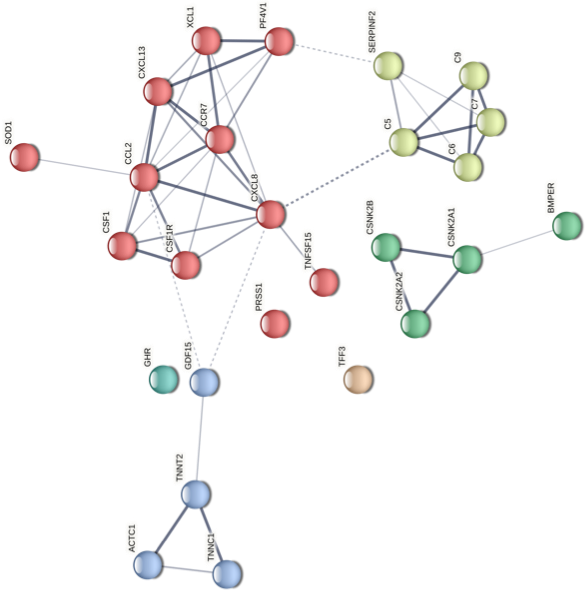


### Supplementary Tables

Table S1: Covariate availability and platform in each cohort (Y=available, N=not available). * smoking status coded as 0=never, 1=former, 2=current. MMRC = modified medical council research. FEV1 = forced expiratory volume in 1 second.

| *Covariate* | *COPDGene* | *LSC* | *MESA* | *SPIROMICS* |
| --- | --- | --- | --- | --- |
| age | Y | Y | Y | Y |
| sex | Y | Y | Y | Y |
| pack-years of smoking | Y | Y | Y | Y |
| smoking status* | Y | Y | Y | Y |
| 6-minute walk distance | Y | N | N | Y |
| body-mass index | Y | Y | Y | Y |
| FEV1 (L) | Y | Y | Y | Y |
| MMRC dyspnea score | Y | Y | N | Y |
| SomaScan 1.3K | Y | N | Y | Y |
| SomaScan 5K | Y (at 5-year follow up) | Y | N | N |

Table S2. Hazard ratios for protein risk scores (protRSs) calculated by multiple methods and tested in the COPDGene testing set (n=550). Models were adjusted for age, sex, self-identified race, current smoking status, pack years of smoking, FEV_1_ % predicted, MMRC dyspnea score, BMI, and 6-minute walk distance. LASSO = least absolute shrinkage and selection operater. ada-LASSO = adaptive LASSO. RSF = random survival forest. MMRC= Modified medical research council. FEV_1_ = forced expiratory volume in 1 second. COPDGene = Genetic Epidemiology of COPD study. Bonferroni threshold: 0.05/4 = 0.0125.

| *Score* | *No. proteins* | *unadj. HR (95% CI)* | *p (unadj.)* | *adj. HR (95% CI)* | *p (adj.)* |
| --- | --- | --- | --- | --- | --- |
| ProtRS LASSO | 15 | 3.5 (2.7 - 4.5) | 5.00E-21 | 2.7 (1.9 - 3.7) | 3.00E-09 |
| ProtRS ada-LASSO | 100 | 2.6 (2.1 - 3.3) | 8.90E-16 | 2 (1.4 - 2.7) | 1.80E-05 |
| ProtRS Random Forest | 100 | 2.5 (1.9 - 3.2) | 8.20E-13 | 2.4 (1.7 - 3.3) | 1.30E-07 |
| ProtRS RSF | 30 | 2.2 (1.7 - 2.8) | 1.70E-10 | 1.8 (1.3 - 2.3) | 4.70E-05 |

Table S3. Weights, protein symbols and names of the proteins included in the protRS (LASSO). Proteins are sorted in descending order by absolute effect size.

| *Weight* | *Entrez Gene Symbol* | *Target full name* |
| --- | --- | --- |
| -1.207925802 | SERPINF2 | Alpha-2-antiplasmin |
| 0.726874752 | C7 | Complement component C7 |
| 0.42639822 | TNNT2 | Troponin-T |
| -0.34513894 | GHR | Growth hormone receptor |
| 0.211008236 | BMPER | BMP-binding endothelial regulator protein |
| 0.19345671 | CXCL13 | C-X-C motif chemokine 13 |
| 0.176034565 | GDF15 | Growth/differentiation factor 15 |
| 0.158443283 | CSNK2A2 CSNK2B | Casein kinase II 2-alpha':2-beta heterotetramer |
| 0.142688432 | SOD1 | Superoxide dismutase [Cu-Zn] |
| -0.142110098 | PRSS1 | Trypsin-1 |
| 0.131709691 | CSF1 | Macrophage colony-stimulating factor 1 |
| 0.120898241 | TNFSF15 | Tumor necrosis factor ligand superfamily member 15 |
| 0.098935599 | TFF3 | Trefoil factor 3 |
| 0.048425179 | XCL1 | Lymphotactin |
| 0.005542167 | CXCL8 | Interleukin-8 |

Table S4: Area-under-the-receiver-operating-characteristic (AUC) for prediction of death using models with a protein risk score (protRS), a clinical model (full model: age, sex, self-reported race, current smoking status, pack years of smoking (when available), FEV_1_% predicted, BMI, MMRC dyspnea score, and 6-minute walk distance, as available; reduced model: age, sex, self-reported race, pack years of smoking), and the protRS + clinical model. See Methods for details about which variables are available in specific cohorts. P-values are DeLong p-values which are used to compare model AUCs.

| *Measure* | **Full Model** | | | | **Reduced Model** | | | |
| --- | --- | --- | --- | --- | --- | --- | --- | --- |
|  | *COPDGene testing set* | *LSC* | *MESA* | *SPIROMICS* | *COPDGene testing set* | *LSC* | *MESA* | *SPIROMICS* |
| AUC [ProtRS] | 0.81 | 0.68 | 0.75 | 0.73 | 0.81 | 0.68 | 0.75 | 0.73 |
| AUC [Clinical model] | 0.84 | 0.85 | 0.77 | 0.83 | 0.82 | 0.82 | 0.76 | 0.6 |
| AUC [ProtRS + clinical model] | 0.88 | 0.86 | 0.8 | 0.85 | 0.87 | 0.84 | 0.79 | 0.74 |
| p (clinical vs. protRS) | 0.33 | 0.0024 | 0.49 | 0.059 | 0.81 | 0.018 | 0.89 | 0.013 |
| p (Combined vs clinical) | 0.029 | 0.38 | 0.11 | 0.33 | 0.0052 | 0.13 | 0.09 | 0.0046 |

Table S5. Multivariable Cox regressions were performed to test the association of each protein in the protein risk score with mortality in the COPDGene testing set, LSC, MESA, and SPIROMICS. MESA was missing the CSF1 protein. All other cohorts had all 15 proteins in their assay, though LSC used the Somascan 5K platform. Inverse variance fixed effects meta-analysis adjusted hazard ratios I 95% confidence intervals and I2 values are shown in the right-hand columns. The table is organized from lowest to highest I2 values. Models were adjusted for age, sex, self-identified race, current smoking status, pack years of smoking, FEV1 % predicted, MMRC dyspnea score, BMI, and 6-minute walk distance. See Table 1 legend for other abbreviations. * = false discovery rate (FDR) p-value < 0.05. † = p-value < 0.05 in meta-analysis.

| *Somascan aptamer* | *Entrez Gene Symbol* | **COPDGene testing set** | | **LSC** | | **MESA** | | **SPIROMICS** | | **Meta-analysis** | **I^2^** |
| --- | --- | --- | --- | --- | --- | --- | --- | --- | --- | --- | --- |
|  |  | *HR (95% CI)* | *p* | *HR (95% CI)* | *p* | *HR (95% CI)* | *p* | *HR (95% CI)* | *p* | *HR (95% CI)* |  |
| 2794_60_2 | SOD1 | 1.2 (0.61 - 2.2) | 0.66 | 0.5 (0.23 - 1.1) | 0.084 | 0.8 (0.46 - 1.4) | 0.43 | 1.1 (0.3 - 4) | 0.88 | 0.83 (0.583 - 1.19) | 0 |
| 2948_58_2 | GHR | 0.31 (0.14 - 0.69) | 0.0046 | 0.45 (0.19 - 1.1) | 0.068 | 0.28 (0.15 - 0.54) | 0.00013* | 0.58 (0.2 - 1.7) | 0.33 | 0.35 (0.234 - 0.529)† | 0 |
| 3487_32_2 | CXCL13 | 1.9 (1.3 - 2.7) | 0.0018 | 1.9 (1.2 - 3) | 0.01 | 1.3 (0.8 - 2.3) | 0.27 | 1.6 (0.85 - 2.9) | 0.15 | 1.7 (1.33 - 2.15)† | 0 |
| 3738_54_4 | CSF1 | 1.3 (0.51 - 3.5) | 0.55 | 1.3 (0.63 - 2.6) | 0.5 | NA | NA | 1.4 (0.55 - 3.7) | 0.46 | 1.3 (0.815 - 2.18) | 0 |
| 4374_45_2 | GDF15 | 5.1 (2.6 - 9.7) | 1.10E-06* | 3.5 (1.9 - 6.7) | 0.00012* | 3.1 (1.9 - 5.1) | 7.90E-06* | 1.9 (0.78 - 4.8) | 0.16 | 3.4 (2.48 - 4.66)† | 0.013 |
| 3654_27_5 | BMPER | 1.3 (0.65 - 2.7) | 0.43 | 0.5 (0.17 - 1.5) | 0.22 | 0.59 (0.3 - 1.2) | 0.14 | 1.1 (0.6 - 2.2) | 6.80E-01 | 0.9 (0.621 - 1.3) | 0.29 |
| 4143_74_2 | XCL1 | 1.3 (0.95 - 1.9) | 0.099 | 0.44 (0.13 - 1.6) | 0.21 | 1.5 (0.73 - 3.1) | 0.27 | 0.71 (0.31 - 1.6) | 0.4 | 1.2 (0.901 - 1.57) | 0.37 |
| 4721_54_2 | TFF3 | 2.3 (1.3 - 4) | 0.0052 | 1.6 (1 - 2.6) | 0.042 | 3.7 (2.2 - 6.3) | 1.60E-06* | 2 (0.54 - 7.4) | 0.3 | 2.3 (1.7 - 3.03)† | 0.44 |
| 5315_22_3 | TNNT2 | 2.3 (1.3 - 3.9) | 0.0035 | 0.55 (0.2 - 1.5) | 0.26 | 1.5 (1.1 - 2.1) | 0.01 | 2.2 (1.2 - 3.8) | 0.0087 | 1.7 (1.3 - 2.1)† | 0.56 |
| 3447_64_2 | CXCL8 | 2.7 (1.3 - 5.7) | 0.0073 | 1.2 (0.81 - 1.8) | 0.35 | 0.88 (0.5 - 1.6) | 0.67 | 1.7 (1.2 - 2.4) | 0.0018 | 1.5 (1.16 - 1.82)† | 0.6 |
| 3024_18_2 | SERPINF2 | 0.14 (0.017 - 1.1) | 0.064 | 0.26 (0.045 - 1.5) | 0.12 | 0.094 (0.015 - 0.6) | 0.013 | 7.1 (0.55 - 92) | 0.13 | 0.28 (0.102 - 0.751)† | 0.62 |
| 5226_36_3 | CSNK2A2 CSNK2B | 0.98 (0.58 - 1.7) | 9.30E-01 | 0.82 (0.59 - 1.2) | 0.26 | 1.5 (1.1 - 2) | 6.00E-03 | 0.49 (0.23 - 1) | 0.059 | 1.1 (0.878 - 1.3) | 0.75 |
| 2888_49_2 | C7 | 3.7 (1.8 - 7.6) | 0.00027* | 0.74 (0.35 - 1.6) | 0.43 | 4.5 (1.7 - 12) | 2.80E-03 | 2.5 (0.6 - 10) | 0.21 | 2.1 (1.39 - 3.31)† | 0.76 |
| 2968_61_1 | TNFSF15 | 2.5 (1.3 - 4.7) | 0.0044 | 0.78 (0.41 - 1.5) | 0.45 | 2.7 (1.8 - 4) | 2.60E-06* | 0.6 (0.23 - 1.6) | 0.31 | 1.8 (1.35 - 2.41)† | 0.81 |
| 3049_61_2 | PRSS1 | 0.79 (0.44 - 1.4) | 0.42 | 1.3 (0.8 - 2) | 0.31 | 1.7 (1.1 - 2.9) | 0.031 | 0.45 (0.31 - 0.65) | 2.30E-05* | 0.84 (0.667 - 1.06) | 0.86 |

Table S6. Multivariable logistic regression models were used to test the association of different protein risk score (protRS) modelling approaches with cause-specific mortality in the COPDGene testing set (n=550). Models were adjusted for age, sex, self-identified race, current smoking status, pack years of smoking, FEV1 % predicted, MMRC dyspnea score, BMI, and 6-minute walk distance. LASSO = least absolute shrinkage and selection operater. ada-LASSO = adaptive LASSO. RSF = random survival forest. MMRC= Modified medical research council. FEV1 = forced expiratory volume in 1 second. COPDGene = Genetic Epidemiology of COPD study. AUC=area-under-the-receiver-operating-characteristic-curve. OR=odds ratio. DeLong p-values were used to compare model AUCs.

| *Cause-specific mortality* | *events* | *score* | *OR (95% CI)* | *p* | *AUC [protRS]* | *AUC [clinical model]* | *AUC [clinical model + protRS]* | *p [AUC (clinical model + protRS) vs. AUC (clinical model)]* |
| --- | --- | --- | --- | --- | --- | --- | --- | --- |
| Respiratory/COPD | 25 | ProtRS LASSO | 9.2 (3.2 - 26) | 3.60E-05 | 0.88 | 0.94 | 0.97 | 0.064 |
| Respiratory/COPD | 25 | ProtRS ada-LASSO | 4.6 (1.9 - 12) | 0.00097 | 0.83 | 0.94 | 0.96 | 0.1 |
| Respiratory/COPD | 25 | ProtRS Random Forest | 5.7 (2.3 - 14) | 0.00017 | 0.77 | 0.94 | 0.97 | 0.16 |
| Respiratory/COPD | 25 | ProtRS RSF | 3.6 (1.8 - 7.3) | 0.00043 | 0.81 | 0.94 | 0.96 | 0.11 |
| Cardiovascular | 8 | ProtRS LASSO | 3.6 (1.1 - 11) | 0.029 | 0.87 | 0.92 | 0.94 | 0.041 |
| Cardiovascular | 8 | ProtRS ada-LASSO | 2.4 (0.94 - 6.1) | 0.069 | 0.77 | 0.92 | 0.93 | 0.46 |
| Cardiovascular | 8 | ProtRS Random Forest | 3.3 (1.2 - 9) | 0.023 | 0.81 | 0.92 | 0.94 | 0.012 |
| Cardiovascular | 8 | ProtRS RSF | 2.3 (0.96 - 5.3) | 0.062 | 0.79 | 0.92 | 0.94 | 0.13 |

Table S7: Multivariable logistic regressions were performed for the ProtRS, the Ganz cardiovascular mortality protein score, and the residuals of the Ganz score and ProtRS in the COPDGene testing set. Models were adjusted for age, sex, self-identified race, current smoking status, pack years of smoking, FEV1 % predicted, MMRC dyspnea score, BMI, and 6-minute walk distance. See Table 1 legend for other abbreviations.

| *outcome* | *predictor* | *OR (95% CI)* | *p* |
| --- | --- | --- | --- |
| All-cause mortality | Protein Risk Score | 3.3 (2.13 to 4.97) | 5.00E-08 |
| All-cause mortality | Protein Risk Score (Ganz residuals) | 1.9 (1.23 to 3.06) | 0.0045 |
| All-cause mortality | Ganz CV Mortality Score | 2.4 (1.67 to 3.47) | 2.40E-06 |
| Respiratory mortality | Protein Risk Score | 9.3 (3.31 to 26.3) | 2.40E-05 |
| Respiratory mortality | Protein Risk Score (Ganz residuals) | 4.2 (1.77 to 10.1) | 0.0012 |
| Respiratory mortality | Ganz CV Mortality Score | 3 (1.45 to 6.39) | 0.0032 |
| Cardiovascular mortality | Protein Risk Score | 4.8 (1.57 to 14.6) | 0.0059 |
| Cardiovascular mortality | Protein Risk Score (Ganz residuals) | 1.7 (0.598 to 4.84) | 0.32 |
| Cardiovascular mortality | Ganz CV Mortality Score | 4.8 (1.74 to 13.2) | 0.0024 |

Table S8. Reactome pathway analyses. Proteins included in the protein risk score were used as inputs into STRING and mapped to the human protein-protein interactome (5 interactors in first shell, 5 interactors in second shell). Pathway enrichement analyses were performed on the resulting protein-protein interaction network. Only pathways with FDR p-values < 0.05 were included in this table.

| *Reactome ID* | *term description* | *observed gene count* | *background gene count* | *strength* | *false discovery rate* |
| --- | --- | --- | --- | --- | --- |
| HSA-166665 | Terminal pathway of complement | 4 | 8.00E+00 | 2.58 | 2.66E-06 |
| HSA-380108 | Chemokine receptors bind chemokines | 5 | 57 | 1.82 | 1.86E-05 |
| HSA-375276 | Peptide ligand-binding receptors | 6 | 194 | 1.37 | 0.00015 |
| HSA-201688 | WNT mediated activation of DVL | 3 | 8 | 2.45 | 0.00019 |
| HSA-2514853 | Condensation of Prometaphase Chromosomes | 3 | 11 | 2.32 | 0.0003 |
| HSA-8934903 | Receptor Mediated Mitophagy | 3 | 11 | 2.32 | 0.0003 |
| HSA-977606 | Regulation of Complement cascade | 4 | 49 | 1.79 | 0.0003 |
| HSA-168256 | Immune System | 12 | 1979 | 0.66 | 0.00069 |
| HSA-445144 | Signal transduction by L1 | 3 | 21 | 2.03 | 0.00086 |
| HSA-1483191 | Synthesis of PC | 3 | 28 | 1.91 | 0.0017 |
| HSA-390522 | Striated Muscle Contraction | 3 | 36 | 1.8 | 0.0029 |
| HSA-6814122 | Cooperation of PDCL (PhLP1) and TRiC/CCT in G-protein beta folding | 3 | 38 | 1.78 | 0.003 |
| HSA-8939243 | RUNX1 interacts with co-factors whose precise effect on RUNX1 targets is not known | 3 | 37 | 1.79 | 0.003 |
| HSA-1280215 | Cytokine Signaling in Immune system | 7 | 706 | 0.88 | 0.0036 |
| HSA-6783783 | Interleukin-10 signaling | 3 | 45 | 1.7 | 0.0041 |
| HSA-8948751 | Regulation of PTEN stability and activity | 3 | 68 | 1.52 | 0.0125 |
| HSA-9711123 | Cellular response to chemical stress | 4 | 195 | 1.19 | 0.0137 |
| HSA-162582 | Signal Transduction | 11 | 2540 | 0.52 | 0.0196 |
| HSA-6804756 | Regulation of TP53 Activity through Phosphorylation | 3 | 92 | 1.39 | 0.0239 |
| HSA-449147 | Signaling by Interleukins | 5 | 453 | 0.92 | 0.0245 |
| HSA-2262752 | Cellular responses to stress | 6 | 747 | 0.78 | 0.0286 |
| HSA-9755511 | KEAP1-NFE2L2 pathway | 3 | 105 | 1.34 | 0.0288 |
| HSA-449836 | Other interleukin signaling | 2 | 24 | 1.8 | 0.038 |
| HSA-380994 | ATF4 activates genes in response to endoplasmic reticulum stress | 2 | 27 | 1.75 | 0.0446 |
| HSA-418594 | G alpha (i) signalling events | 4 | 309 | 0.99 | 0.0448 |

Table S9: MCL clusters based on the protein risk score. Proteins included in the protein risk score were used as inputs into STRING and mapped to the human protein-protein interactome (5 interactors in first shell, 5 interactors in second shell). MCL clustering with an inflation factor of 3 was performed on the resulting network.

| *cluster color* | *gene count* | *protein name* | *protein identifier* | *protein description* |
| --- | --- | --- | --- | --- |
| Red | 9 | CXCL1 | 9606.ENSP00000379110 | Growth-regulated alpha protein; Has chemotactic activity for neutrophils. May play a role in inflammation and exerts its effects on endothelial cells in an autocrine fashion. In vitro, the processed forms GRO- alpha(4-73), GRO-alpha(5-73) and GRO-alpha(6-73) show a 30-fold higher chemotactic activity; Chemokine ligands |
| Red | 9 | CXCL13 | 9606.ENSP00000286758 | C-X-C motif chemokine 13; Chemotactic for B-lymphocytes but not for T-lymphocytes, monocytes and neutrophils. Does not induce calcium release in B- lymphocytes. Binds to BLR1/CXCR5; Chemokine ligands |
| Red | 9 | CXCL2 | 9606.ENSP00000427279 | C-X-C motif chemokine 2; Produced by activated monocytes and neutrophils and expressed at sites of inflammation. Hematoregulatory chemokine, which, in vitro, suppresses hematopoietic progenitor cell proliferation. GRO-beta(5-73) shows a highly enhanced hematopoietic activity |
| Red | 9 | CXCL5 | 9606.ENSP00000296027 | C-X-C motif chemokine 5; Involved in neutrophil activation. In vitro, ENA-78(8- 78) and ENA-78(9-78) show a threefold higher chemotactic activity for neutrophil granulocytes; Chemokine ligands |
| Red | 9 | CXCL8 | 9606.ENSP00000306512 | Interleukin-8; IL-8 is a chemotactic factor that attracts neutrophils, basophils, and T-cells, but not monocytes. It is also involved in neutrophil activation. It is released from several cell types in response to an inflammatory stimulus. IL-8(6-77) has a 5-10-fold higher activity on neutrophil activation, IL-8(5-77) has increased activity on neutrophil activation and IL-8(7-77) has a higher affinity to receptors CXCR1 and CXCR2 as compared to IL-8(1-77), respectively; Chemokine ligands |
| Red | 9 | CXCR1 | 9606.ENSP00000295683 | C-X-C chemokine receptor type 1; Receptor to interleukin-8, which is a powerful neutrophils chemotactic factor. Binding of IL-8 to the receptor causes activation of neutrophils. This response is mediated via a G-protein that activate a phosphatidylinositol-calcium second messenger system. This receptor binds to IL-8 with a high affinity and to MGSA (GRO) with a low affinity; C-X-C motif chemokine receptors |
| Red | 9 | CXCR2 | 9606.ENSP00000319635 | C-X-C chemokine receptor type 2; Receptor for interleukin-8 which is a powerful neutrophil chemotactic factor. Binding of IL-8 to the receptor causes activation of neutrophils. This response is mediated via a G-protein that activates a phosphatidylinositol-calcium second messenger system. Binds to IL-8 with high affinity. Also binds with high affinity to CXCL3, GRO/MGSA and NAP-2 |
| Red | 9 | XCL1 | 9606.ENSP00000356792 | Lymphotactin; Chemotactic activity for lymphocytes but not for monocytes or neutrophils. In thymus, mediates medullary accumulation of thymic dendritic cells and contributes to regulatoy T cell development, playing a role in self-tolerance establishment; Chemokine ligands |
| Red | 9 | XCR1 | 9606.ENSP00000310405 | Chemokine XC receptor 1; Receptor for chemokines SCYC1 and SCYC2. Subsequently transduces a signal by increasing the intracellular calcium ions level. Receptor for XCL1/Lymphotactin; X-C motif chemokine receptors |
| Brown | 4 | GDF15 | 9606.ENSP00000252809 | Growth differentiation factor 15; Belongs to the TGF-beta family |
| Brown | 4 | TNNC1 | 9606.ENSP00000232975 | Troponin C, slow skeletal and cardiac muscles; Troponin is the central regulatory protein of striated muscle contraction. Tn consists of three components: Tn-I which is the inhibitor of actomyosin ATPase, Tn-T which contains the binding site for tropomyosin and Tn-C. The binding of calcium to Tn-C abolishes the inhibitory action of Tn on actin filaments; EF-hand domain containing |
| Brown | 4 | TNNI3 | 9606.ENSP00000341838 | Troponin I, cardiac muscle; Troponin I is the inhibitory subunit of troponin, the thin filament regulatory complex which confers calcium-sensitivity to striated muscle actomyosin ATPase activity |
| Brown | 4 | TNNT2 | 9606.ENSP00000236918 | Troponin T, cardiac muscle; Troponin T is the tropomyosin-binding subunit of troponin, the thin filament regulatory complex which confers calcium-sensitivity to striated muscle actomyosin ATPase activity |
| Olive | 4 | GH1 | 9606.ENSP00000312673 | Somatotropin; Plays an important role in growth control. Its major role in stimulating body growth is to stimulate the liver and other tissues to secrete IGF-1. It stimulates both the differentiation and proliferation of myoblasts. It also stimulates amino acid uptake and protein synthesis in muscle and other tissues; Endogenous ligands |
| Olive | 4 | GHR | 9606.ENSP00000483403 | Growth hormone receptor; Receptor for pituitary gland growth hormone involved in regulating postnatal body growth. On ligand binding, couples to the JAK2/STAT5 pathway (By similarity); Belongs to the type I cytokine receptor family. Type 1 subfamily |
| Olive | 4 | JAK2 | 9606.ENSP00000371067 | Tyrosine-protein kinase JAK2; Non-receptor tyrosine kinase involved in various processes such as cell growth, development, differentiation or histone modifications. Mediates essential signaling events in both innate and adaptive immunity. In the cytoplasm, plays a pivotal role in signal transduction via its association with type I receptors such as growth hormone (GHR), prolactin (PRLR), leptin (LEPR), erythropoietin (EPOR), thrombopoietin (THPO); or type II receptors including IFN-alpha, IFN-beta, IFN-gamma and multiple interleukins. Following ligand-binding to cell surface receptors, [...] |
| Olive | 4 | TFF3 | 9606.ENSP00000430690 | Trefoil factor 3; Involved in the maintenance and repair of the intestinal mucosa. Promotes the mobility of epithelial cells in healing processes (motogen) |
| Green | 3 | CSNK2A1 | 9606.ENSP00000217244 | Casein kinase II subunit alpha; Catalytic subunit of a constitutively active serine/threonine-protein kinase complex that phosphorylates a large number of substrates containing acidic residues C-terminal to the phosphorylated serine or threonine. Regulates numerous cellular processes, such as cell cycle progression, apoptosis and transcription, as well as viral infection. May act as a regulatory node which integrates and coordinates numerous signals leading to an appropriate cellular response. During mitosis, functions as a component of the p53/TP53-dependent spindle assembly checkpoin [...] |
| Green | 3 | CSNK2A2 | 9606.ENSP00000262506 | Casein kinase II subunit alpha; Catalytic subunit of a constitutively active serine/threonine-protein kinase complex that phosphorylates a large number of substrates containing acidic residues C-terminal to the phosphorylated serine or threonine. Regulates numerous cellular processes, such as cell cycle progression, apoptosis and transcription, as well as viral infection. May act as a regulatory node which integrates and coordinates numerous signals leading to an appropriate cellular response. During mitosis, functions as a component of the p53/TP53-dependent spindle assembly checkpoin [...] |
| Green | 3 | CSNK2B | 9606.ENSP00000365042 | Casein kinase II subunit beta; Participates in Wnt signaling (By similarity). Plays a complex role in regulating the basal catalytic activity of the alpha subunit; Belongs to the casein kinase 2 subunit beta family |
| Blue | 3 | CSF1 | 9606.ENSP00000327513 | Macrophage colony-stimulating factor 1; Cytokine that plays an essential role in the regulation of survival, proliferation and differentiation of hematopoietic precursor cells, especially mononuclear phagocytes, such as macrophages and monocytes. Promotes the release of proinflammatory chemokines, and thereby plays an important role in innate immunity and in inflammatory processes. Plays an important role in the regulation of osteoclast proliferation and differentiation, the regulation of bone resorption, and is required for normal bone development. Required for normal male and female [...] |
| Blue | 3 | CSF1R | 9606.ENSP00000286301 | Macrophage colony-stimulating factor 1 receptor; Tyrosine-protein kinase that acts as cell-surface receptor for CSF1 and IL34 and plays an essential role in the regulation of survival, proliferation and differentiation of hematopoietic precursor cells, especially mononuclear phagocytes, such as macrophages and monocytes. Promotes the release of proinflammatory chemokines in response to IL34 and CSF1, and thereby plays an important role in innate immunity and in inflammatory processes. Plays an important role in the regulation of osteoclast proliferation and differentiation, the regulat [...] |
| Blue | 3 | IL34 | 9606.ENSP00000397863 | Interleukin-34; Cytokine that promotes the proliferation, survival and differentiation of monocytes and macrophages. Promotes the release of proinflammatory chemokines, and thereby plays an important role in innate immunity and in inflammatory processes. Plays an important role in the regulation of osteoclast proliferation and differentiation, and in the regulation of bone resorption. Signaling via CSF1R and its downstream effectors stimulates phosphorylation of MAPK1/ERK2 AND MAPK3/ERK1; Belongs to the IL-34 family |
| Light Sky Blue | 3 | TNFRSF25 | 9606.ENSP00000367013 | Tumor necrosis factor receptor superfamily member 25; Receptor for TNFSF12/APO3L/TWEAK. Interacts directly with the adapter TRADD. Mediates activation of NF-kappa-B and induces apoptosis. May play a role in regulating lymphocyte homeostasis; Tumor necrosis factor receptor superfamily |
| Light Sky Blue | 3 | TNFRSF6B | 9606.ENSP00000359013 | Tumor necrosis factor receptor superfamily, member 6b, decoy; Decoy receptor that can neutralize the cytotoxic ligands TNFS14/LIGHT, TNFSF15 and TNFSF6/FASL. Protects against apoptosis; Tumor necrosis factor receptor superfamily |
| Light Sky Blue | 3 | TNFSF15 | 9606.ENSP00000363157 | Tumor necrosis factor ligand superfamily member 15; Receptor for TNFRSF25 and TNFRSF6B. Mediates activation of NF-kappa-B. Inhibits vascular endothelial growth and angiogenesis (in vitro). Promotes activation of caspases and apoptosis; Belongs to the tumor necrosis factor family |
| Medium Blue | 2 | PRSS1 | 9606.ENSP00000308720 | Trypsin-1; Has activity against the synthetic substrates Boc-Phe- Ser-Arg-Mec, Boc-Leu-Thr-Arg-Mec, Boc-Gln-Ala-Arg-Mec and Boc-Val- Pro-Arg-Mec. The single-chain form is more active than the two- chain form against all of these substrates; Belongs to the peptidase S1 family |
| Medium Blue | 2 | SERPINF2 | 9606.ENSP00000321853 | Alpha-2-antiplasmin; Serine protease inhibitor. The major targets of this inhibitor are plasmin and trypsin, but it also inactivates matriptase-3/TMPRSS7 and chymotrypsin; Serpin peptidase inhibitors |

Table S10: Enrichr analysis of Multi-marker Analysis of GenoMic Annotation (MAGMA) diseases and drugs database using proteins in the protein risk score as inputs. Enrichment terms with associated q-values less than 0.05 are included. NA - not applicable.

| term | p-value | q-value |
| --- | --- | --- |
| Pamidronate | 3.99E-03 | 2.76E-02 |
| Glucocorticoid Receptor Antagonist | 5.59E-03 | 2.76E-02 |
| Aminoglutethimide | 5.59E-03 | 2.76E-02 |
| Calcitriol | 7.97E-03 | 2.76E-02 |
| Nafamostat | 8.77E-03 | 2.76E-02 |
| Naproxen | 9.56E-03 | 2.76E-02 |
| Clarithromycin | 1.04E-02 | 2.76E-02 |
| Leflunomide | 1.11E-02 | 2.76E-02 |
| Dacarbazine | 1.11E-02 | 2.76E-02 |
| Dihydroorotate Dehydrogenase Inhibitor | 1.11E-02 | 2.76E-02 |
| PDGFR Tyrosine Kinase Receptor Inhibitor | 1.19E-02 | 2.76E-02 |
| VEGFR Inhibitor | 1.19E-02 | 2.76E-02 |
| Axitinib | 1.19E-02 | 2.76E-02 |
| Danazol | 1.27E-02 | 2.76E-02 |
| Melanin Inhibitor | 1.35E-02 | 2.76E-02 |
| Hydroquinone | 1.35E-02 | 2.76E-02 |
| Methimazole | 1.43E-02 | 2.81E-02 |
| Lansoprazole | 2.06E-02 | 3.90E-02 |
| Diclofenac | 2.45E-02 | 4.18E-02 |
| Retinol | 2.45E-02 | 4.18E-02 |
| Retinoid Receptor Ligand | 2.45E-02 | 4.18E-02 |
| Omeprazole | 2.69E-02 | 4.18E-02 |
| Ribavirin | 2.84E-02 | 4.18E-02 |
| Pentoxifylline | 2.84E-02 | 4.18E-02 |
| Paracetamol | 3.00E-02 | 4.18E-02 |
| Tretinoin | 3.00E-02 | 4.18E-02 |
| Sunitinib | 3.39E-02 | 4.60E-02 |
| Verapamil | 3.70E-02 | 4.90E-02 |
| Colchicine | 3.85E-02 | 4.96E-02 |
| Etoposide | 3.93E-02 | 4.96E-02 |

## References

1. Candia, J. *et al.* Assessment of Variability in the SOMAscan Assay. *Sci Rep* **7**, 14248 (2017).

2. Serban, K. A. *et al.* Unique and shared systemic biomarkers for emphysema in Alpha-1 Antitrypsin deficiency and chronic obstructive pulmonary disease. *EBioMedicine* **84**, 104262 (2022).

3. McGarvey, L. P., John, M., Anderson, J. A., Zvarich, M. & Wise, R. A. Ascertainment of cause‐specific mortality in COPD: operations of the TORCH Clinical Endpoint Committee. *Thorax* **62**, 411–415 (2007).

4. Ngo, D. *et al.* Systemic Markers of Lung Function and Forced Expiratory Volume in 1 Second Decline across Diverse Cohorts. *Ann Am Thorac Soc* **20**, 1124–1135 (2023).

5. Taliun, D. *et al.* Sequencing of 53,831 diverse genomes from the NHLBI TOPMed Program. *Nature* **590**, 290–299 (2021).

6. Post, W. S. *et al.* Racial and Ethnic Differences in All-Cause and Cardiovascular Disease Mortality: The MESA Study. *Circulation* **146**, 229–239 (2022).
